# Investigating the market for cultivated meat as pet food: A survey analysis

**DOI:** 10.1371/journal.pone.0275009

**Published:** 2022-12-30

**Authors:** Alice Oven, Barbara Yoxon, Josh Milburn

**Affiliations:** 1 Faculty of Health and Wellbeing, University of Winchester, Winchester, United Kingdom; 2 Department of Politics, Philosophy and Religion, University of Lancaster, Lancaster, United Kingdom; 3 International Relations, Politics and History, Loughborough University, Loughborough, United Kingdom; University of Florida, UNITED STATES

## Abstract

The number of people reducing their meat consumption due to ethical and environmental concerns is growing. However, meat reducers sometimes care for omnivorous or carnivorous pets, creating the ‘vegetarian’s dilemma’. Some meat-reducers opt to feed plant-based diets to companion animals, but others express reservations. Cultivated meat offers a possible third path, but consumer perceptions of cultivated meat as pet food have received little scholarly attention. Using survey data from 729 respondents, we analyzed consumers’ willingness to feed cultivated meat to companion animals, particularly with reference to their own current dietary practices, and their own willingness to eat cultivated meat. Though not all our respondents willing to eat cultivated meat were willing to feed it to their companions, a large majority were (81.4%, 193/237). However, for those unwilling to eat cultivated meat, the story was more complicated. Vegans and vegetarians were less likely to say they would eat cultivated meat (16.4%, 39/238) than meat-eating respondents (40.3%, 198/491). However, among vegans and vegetarians who would not consume cultivated meat, the majority (55.9%, 86/154) indicated that they *would* still feed it to their pets. Among meat-eating respondents, only a small minority (9.6%, 11/114) unwilling to eat cultivated meat would feed it to their pets. Consequently, we suggest that the potential market for cultivated meat for pet food is markedly different from the potential market for cultivated meat from human consumption. A key concern among our respondents about feeding cultivated meat to pets was a worry that it was not healthy, indicating that there may be easy gains in cultivated pet food’s uptake through messaging relating to safety and nutritional completeness.

## Introduction

Animal agriculture is one of the leading causes of climate change, biodiversity loss, and water pollution [1]. At the same time, the COVID-19 pandemic has brought intensive animal agriculture into sharp focus as a major driver of human health crises, including future zoonotic pandemics and the development of antibiotic-resistant strains of bacteria [2]. Animal agriculture is also directly responsible for the death of tens of billions of terrestrial vertebrates a year [3]. Many of these animals, an estimated 99% in the United States [4], are raised in intensive operations and subject to considerable suffering. Both the death and the suffering of these animals raise serious ethical concerns from a wide range of perspectives [5]. Despite these assorted challenges, research suggests that many individuals–especially those committed to meat-eating–are not open to solutions that challenge intensive agriculture. For example, Dhont, Piazza, and Hodson [6] found that meat-eating played a role in the ‘willful disregard’ of factory farming as a catalyst for pandemics.

While the negative consequences of animal agriculture are becoming increasingly familiar [7–9], it is rarely considered that food for humans is not the only driver of intensive farming. Yet conventional, meat-based pet food uses more than a quarter of all animal-derived calories in the United States, where dogs and cats consume 30 percent of all meat from intensively farmed animals [10]. In the United Kingdom (UK), 51 percent of households contained pets in 2020 [11]. There are also signs that pet ownership is on the rise, with over 17 million households in the UK adopting a new companion animal since the start of the COVID pandemic in 2020 [12]. These pets are predominantly dogs and cats, who usually consume meat at least daily.

Calculating the ‘environmental pawprint’ of pet food is not straightforward, especially as some pet food utilizes ‘waste’ products deemed undesirable for human consumption [10, 13, 14]. But the pet-food industry, which is worth around US$100 billion a year [15], provides an important income source for harmful farming industries, making them more profitable [16]. Trends in pet-food production towards so-called ‘human grade’ meat (meat perceived as of a quality suitable for human consumption), as well as potential changes in human dietary practices leading to fewer ‘waste’ animal products, risk exacerbating the impact of pet food by demanding increased farming and slaughter primarily for pet-food production. All of this means that environmentalists concerned about the impacts of diet need to think not only about human dietary practices, but feeding practices, too. There is some evidence that younger pet owners are already doing so: a 2019 pet food industry survey saw 28% of respondents aged 18 to 34 express interest in seeing more sustainably sourced pet food [17].

Similarly, health advocates should be concerned about the impact of the pet-food industry: intensive animal agriculture contributes to the risk of antibiotic-resistant bacteria whether the animals are farmed for human consumption or animal consumption [16]. At the same time, pet feeding practices can be problematic for individuals on ethical grounds. Vegetarians and vegans face what has been called the ‘vegetarian’s dilemma’ [18] or the ‘animal lovers’ paradox’ [19] when deciding what to feed their pets. While they want to do what is best for their pet, they also see a wrong in the slaughter of animals to produce food.

Although a range of recent studies have suggested that plant-based diets are accessible and healthy for both dogs and cats [20–25], other studies have been more critical [26], and the uptake of plant-based feeding of pets has been very limited, even among vegetarians and vegans [27]. Cultivated meat (also known as cell-based meat, cultured meat, *in vitro* meat, clean meat, lab meat, cell meat, and other names) has been touted as part of the solution to the harmful way that animal protein is currently produced for human consumption [28]. In short, cultivated meat is meat grown at the cellular level, rather than the organism level. Beginning with a small cell biopsy, taken from the animal (in principle) without harm or slaughter, so-called cellular agriculturalists can produce meat in bioreactors, without the need to grow a whole animal. Cultivated chicken meat for human consumption is now available in both Singapore and Israel [29], and a range of start-ups are developing further cultivated meat products aimed at a variety of markets. Some of these start-ups are developing cultivated meat specifically for the pet-food market [16, 30]. Companies exploring cultivated meat as pet food include Because Animals, Pristine Pet Food, and Wild Earth in the United States; Appleton Meats in Canada; Good Dog Food in the UK; and Five Letter Foods in Finland.

The possibility of pet food utilizing cultivated meat–hereafter ‘cultivated pet food’, as contrasted with ‘plant-based pet food’ and ‘conventional pet food’–has been championed by some animal ethicists [31], including those skeptical about the production of cultivated meat for human consumption [32], with the acknowledgement that pet food raises different social, ethical, and legal questions to human foods [19]. These might include, for example, different regulatory pathways, reduced ‘yuck’ factor, and differences in nutritional needs between humans and animals.

Given these differences, it has been suggested that cultivated pet food could be a gateway product for the wider cultivated meat industry [16, 33, 34]. Pricing will be a challenge, given consumer expectations that pet food should be cheaper than food for human consumption. However, the possibility of using meats where the donor animal is less expensive to rear, such as mouse, fish, or invertebrates–or meats in which donor animals are not needed at all, because of the existence of immortal cell lines–may help address this. There is, further, less need for cultivated pet food to exactly replicate existing products, meaning its production may be less technologically challenging. There may also be a lower regulatory burden to bring cultivated meat to the pet food market, as the pet-food industry is generally less regulated than the market for human food. As such, though cultivated meat aimed at human consumers has been first to market, it remains possible that widespread availability of cultivated pet food will predate widespread availability of cultivated meat for human consumption.

Despite the potential importance of cultivated pet food as a route to limit the harm of pet food and as a possible steppingstone for the nascent cultivated meat industry, there has been very little social scientific research on consumer perceptions of cultivated pet food. There has been a plethora of studies assessing consumer acceptance of cultivated meat as food for humans [35–43], but we are aware of only one study exploring consumer willingness to feed cultivated meat to pets. This is an Association for Pet Obesity Prevention (APOP) survey, completed by 1,156 pet owners and 574 veterinary professionals in the US [44]. The survey asked pet owners which alternative proteins they would consider feeding their dog or cat. Significantly, ‘clean meat’ was the highest-ranking alternative protein: 55.2 percent of cat owners and 55.8 percent of dog owners reported they would consider feeding clean meat while 27.9 percent of cat and 25.2 percent of dog owners responded ‘Maybe’.

However, these results cannot be taken at face value. The survey also listed ‘cultured poultry’, ‘cultured beef’, ‘cell-based meat’, ‘cultured mouse’ and ‘lab-grown meat’ as separate options, yet these were each supported by less than 25 percent of respondents. The APOP did not give participants any information on what ‘clean meat’ or any of the other options were. The fact that more than twice the number of pet owners would feed ‘clean meat’ to pets than would feed ‘cell-based meat’ or a specific ‘cultured’ meat suggests there may be a misunderstanding of what these foods are. This survey did not unpack these results in relation to the owners’ own diet, nor did it interrogate the reasons why almost half the respondents would not feed ‘clean meat’.

The present study aims to offer the first, preliminary enquiry into consumer willingness to purchase cultivated pet food. Crucially, it explores this in conjunction with consumer’s *own* diets, their willingness to eat cultivated meat themselves, and their current feeding practices. While some existing studies [35, 37] have found that vegetarians and vegans show less willingness to purchase cultivated meat for their own consumption, it is unclear whether a parallel unwillingness exists when it comes to purchasing cultivated pet food. While there is little to be gained environmentally or ethically by persuading vegans and vegetarians to switch from (predominantly) plant-based diets to cultivated meat themselves, there are potential gains in encouraging them to feed cultivated meat to their carnivorous or omnivorous pets, since many do not exclusively feed plant-based pet food [27].

Given that vegans and vegetarians have personally abstained from meat consumption, we assume–drawing on the findings of [27]–that this demographic is most likely to be interested in finding alternatives to conventional pet food. If so, vegans and vegetarians could provide a significant market for cultivated pet food, and even an early uptake market. This would be in sharp contrast to cultivated meat for human consumption, which, based on existing studies, would not find a substantial market among vegans and vegetarians.

Such information will have significant economic value for cultivated pet food startups, as well as established pet food corporations developing alternative proteins for pets. It will also be valuable for advocates and policymakers pursuing the adoption of cellular agriculture to tackle urgent problems with the global food system; not only will uptake of cultivated pet food have value for its own sake, but it could provide a crucial bolster to the wider cellular agriculture industry.

## Methodology and data analysis

### Data collection and sample

Participants were invited to take part in a multiple choice and short answer questionnaire via social media channels. To maximize the number of responses from pet owners with different dietary habits, the questionnaire was distributed among largely vegan (Vegan Runners, University of Winchester Animal Welfare Science) as well as non-vegan (A Life Loved, Burn fitness community, the British Veterinary Nurse Association, Association for Pet Obesity Prevention) social media channels and shared within the first author’s own network of contacts. Respondents were encouraged to share the survey to facilitate a higher response rate and the data was collected between 18 June 2019 and 18 September 2019. Participants were required to be over the age of 16 and own a dog, a cat, or both. Participants who only owned herbivorous pets were excluded from participating in the survey.

Before proceeding with the questionnaire, respondents were informed about the aims of the study (understanding their feeding habits, their attitudes towards both conventional pet food and cultivated or ‘cell-based’ alternatives) and the ethical considerations of the research project. The survey questions included references to ‘cell-based’ meat. However, at the time of submission, the authors decided to use ‘cultivated meat’ to follow the best practice in the field, and all tables and figures will refer to ‘cultivated meat’ rather than ‘cell-based meat’, even if original survey questions and responses referenced ‘cell-based meat’ specifically. All participants gave their informed consent before filling the questionnaire and the study was supported by the research ethics board of the University of Winchester.

The sample included 729 respondents (85% female) with 56% of respondents under the age of 40. Among the respondents, 21.8% identified as vegan, 10.8% as vegetarian, 4.3% as pescatarian, 26.6% as ‘meat-reducer’, and 36.5% as omnivorous. In the survey, ‘omnivore’ was defined as somebody who ‘consumes most animal meats’, and thus as separate from pescetarians and meat-reducers, both of whom may eat fewer meats than self-described omnivores (or aspire to do so). For the purposes of this paper, however, we combined the meat-eating categories, since meat-reducers and pescatarians, unlike vegetarians and vegans, are meat-eaters, and divisions between the meat-consuming groups are much hazier than between meat-consumers, vegetarians, and vegans. Hereafter, then, ‘omnivore’ refers to respondents who consumed any animal meat, including self-described pescatarians and meat-reducers.

The initial questions also recorded participants’ ethnicity (91.8% white), profession, location, and whether they owned a cat (32.7%), a dog (43.5%), or both (23.7%). Table 1 breaks down the demographic profile of the 729 research participants. Country of residence categories were deduced from the open-ended question regarding location, and the participants’ descriptions of their profession allowed extrapolation of jobs explicitly associated with animal care, training, advocacy, or research. Two respondents worked in the pet food industry, and these were given a separate category.

**Table 1:** Sample demographics.

Full sample (N)	729		
Female (%)	85.3	Personal diet (% identifying as…)	
		Omnivore	36.5
Age (%)		Meat-reducer	25.6
16–20	1.2	Vegan	21.8
21–30	25.2	Vegetarian	10.8
31–40	29.8	Pescatarian	4.3
41–50	20.2		
51–60	14.7	Type of pet (%)	
61–70	7.5	Dog(s) only	43.5
71 and over	1.4	Cat(s) only	32.8
		Both dog(s) and cat(s)	23.7
Country of residence (%)			
UK	51.7	Relationship with pet (%)	
USA	19.8	Part of my family	59.1
Australia / NZ	6.0	Like a child to me	28.1
Belgium	5.2	A valued companion	10.3
Netherlands	2.3	A working animal (therapy animal/guide	1.0
Spain	1.8	dog/other)	
France	1.2	Just a pet	1.1
Denmark	1.2	A cute addition to my household	0.4
Finland	1.0	Protection	0.0
Other Europe	3.7		
Canada	2.5	Ethnicity (%)	
India	2.3	White	91.8
UAE	0.4	Mixed / multiple ethnic groups	2.9
South Africa	0.3	Asian/Asian British/Asian American	2.2
China	0.3	Black/African/Caribbean/Black British	0.1
Central / South America	0.1	Other ethnic group	1.2
Japan	0.1	Prefer not to say	1.8
	
Profession (%)		Pet on a medical diet for… (%)	
Non-animal	80.9	No medical diet	88.5
Animal (veterinary/animal nutrition)	7.3	Medical diet (diabetes/weight	11.5
Animal (behaviourist/trainer)	5.1	reduction/renal disease/joint	
Animal (care/charity/advocacy)	4.8	issues/allergies, etc.)	
Animal (scientist/academic)	1.6		
Pet food industry	0.3		

The researchers acknowledge that this sample potentially differs from the general population in several observable ways. First, there was a significant vegan bias: 21.8 percent of our respondents were vegan, while the number in the broader population is much lower. Though precise numbers of vegans are hard to calculate, Bryant [45] reviews 12 representative surveys of dietary habits in the United Kingdom in the 2010s and concludes that the number of vegans is generally estimated at around 1–2% of the adult population, with vegetarians around 2–7%. This coheres a more recent, large-scale representative survey conducted by Ipsos Mori for The Vegan Society, which found that vegans made up 1.2% of the adult population of Great Britain in 2019 [46].

There was also education bias in our sample (generally middle class, affluent professionals), which can be partially attributed to the use of social media sampling [47], and gender bias (85.3 percent female). The gender bias can be explained by the nature of the survey itself. Women are, on average, more likely to be vegetarian or vegan [48], and more likely to own either a cat or a dog [49]. This is compounded by the fact that women are also more likely than men to fill out online surveys [50], and the fact that Facebook users are more likely to be female than the general population [47].

Incidentally, the focus on female attitudes to pet feeding may be justified considering evidence that it is mostly women who will be making the purchasing decision. Industry analysis from GenAnalytics suggests that women make the decision or influence the purchase of 93 percent of food, represent the largest global market opportunity, and are expected to control 75 percent of all household spending by 2028 [51]. Gender analysts Catalyst.org estimate that, on average, 67 percent of all UK household consumption is controlled or influenced by women, and this is much greater in many key household areas like food [51].

Regarding the vegan bias, a National Farmers’ Union report [52] predicted that vegans and vegetarians will make up a quarter of the British population in 2025, with flexitarians just under half of all UK consumers. The US sees a similar pattern: between 2014 and 2017, there was a 600% increase in people identifying as vegans [53]. The vegan demographic represented in this sample may therefore better predict the percentage of vegans in (British) society later in the decade, which could be when cultivated pet food launches in the market. Furthermore, research by Dodd et al. [27] suggests vegans and vegetarians may be more highly represented amongst dog and cat guardians, these groups accounting for 12 percent of their 3,600 sampled pet owners.

It is important to note that all figures and data analysis within this paper should be understood as applying to our sample and will reflect the opinions and attitudes of a mostly female group with a heavy skew towards more middle class, educated consumers who tend to avoid meat consumption. This is likely to generate some unobservable characteristics that differ from the general population (a greater preoccupation with companion animal welfare and/or sustainability, for instance, is possible) that are difficult to control for. Nevertheless, some of our findings, especially those relating to the attitudes among vegans and vegetarian, present valuable findings that could serve as a starting point for future exploration.

The overwhelming majority of respondents identified as white (91.8 percent). Those who were interested enough to complete the questionnaire are likely to have a close relationship with their pet, biasing the data towards those who see their pet as ‘like a child to me’ or ‘part of my family’. It is less likely that those who see their cat or dog as ‘just a pet’ would be willing to give up their time to complete a lengthy questionnaire about their diet. The questionnaire did not include other omnivorous/carnivorous pets, such as reptiles or ferrets, to avoid overcomplicating the data. Further research could help clarify whether owners of such pets might have similar or different responses to owners of dogs and cats.

### Measures

Participants were asked to provide information on their current feeding practices, including whether they fed their pet(s) meat, the frequency of feeding meat, and the type of food they fed to their pet(s) (ready-made branded food, homemade food, raw food). Participants who owned more than one pet were encouraged to describe their divergent feeding practices in an open-ended question. Next, participants were asked about their attitudes to feeding their pet(s) meat and asked to disclose any potential concerns about their practice (health, environmental, animal welfare, or none). Respondents were also asked to describe their attitudes towards cultivated meat and their willingness to feed it to their pet(s), along with the main reasons for wanting to remove conventional meat from the diet of their pet(s). The questionnaire then asked for the owner’s biggest concern about removing conventional meat from the diet of their pet(s), providing Piazza et al.’s [54] 4Ns (meat is ‘necessary’, ‘natural’, ‘normal’, or ‘nice’) as options, as well as potential concerns about price and convenience of cultivated meat. Participants were able to select an option that stated that they had no concerns about removing conventional meat from the diet of their pet(s) and/or provide a more detailed answer in an open-ended question.

Respondents were then provided with a short description of cultivated meat provided by Anderson and Bryant [36]: ‘Clean meat (also called cultured meat or in-vitro meat) is real meat which is grown from animal cells without the need to raise animals. It should not be confused with meat substitutes such as soy, since it is real animal meat: it has the same taste, texture, and the same or better nutritional content as conventionally-produced meat’. After respondents were given this description, they were asked about their attitudes to consuming cultivated meat and chose their primary reservation. An ‘other’ option allowed participants to submit a comment expanding on their perception of cultivated meat for their own consumption. The same set of questions was then asked in relation to their attitudes to feeding their own pet(s) a diet based on cultivated meat. It was stipulated that if the respondent had more than one pet and the pets ate differently, their answer should only apply to their meat-eating pet(s). Respondents were then asked what would most help convince them to feed cultivated meat to their pet(s), with the ‘other’ option allowing for a more nuanced answer.

### Data analysis

We conducted a series of exploratory analyses to investigate the factors affecting the willingness to feed cultivated meat to pets. Our primary goal was to find out whether willingness to feed pets cultivated meat depends on respondents’ own willingness to consume such foods. First, we provide general statistics on attitudes towards cultivated meat and general pet-feeding practices. We then move on to discuss the relationship between the willingness to consume cultivated meat and the willingness to feed it to pets. We discuss the main reservations to feeding cultivated meat and discuss whether respondents’ dietary choices (omnivorous or vegan/vegetarian) affect their decision making.

All data in the survey is categorical, and it was analyzed using Stata statistical software (Release 15. College Station, TX: StataCorp LLC). We used *t*-tests and tests on the equality of proportions to compare attitudes towards cultivated meat for human and pet consumption. More complex relationships are explored in models A1-A2, B1-B7 and C1-C10 in Tables 9–11, which were built using multivariate binary logistic regression. The demographic variables included in all logistic regression models were age, ethnicity, and gender. Other variables in the models included the participant’s diet, type of pet, and their pet’s diet. Variables whose effects were insignificant were not removed from models because their exclusion reduced the fit of the models as determined by AIC scores and the pseudo *R*-squared measure. In Tables 10 and 11, we also include variables recording respondents’ greatest concern about removing meat from the diet of their pet(s), and the greatest concern about feeding cultivated meat to their pet(s). The dependent variable in all models concerned the willingness to feed pet(s) with cultivated pet food, with a binary outcome of ‘yes’ and ‘no/not sure’. Statistical significance was declared at p≤0.05.

## Results

### Who wants to consume cultivated meat?

**Fig 1:**
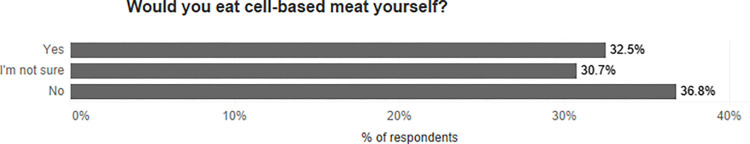
Willingness to try cultivated meat.

As shown in Fig 1, 32.5% of all respondents in the sample were willing to consume cultivated meat. This finding broadly conforms with the results of similar studies carried out in Western countries like Belgium or the US, where acceptance of cultivated meat ranged between 32.9% and 39.3% [41, 42].

The slightly lower percentage in this study is likely due to the relatively high number of vegans and vegetarians surveyed: willingness to eat cultivated meat was much greater among omnivores, where 40.3% of respondents expressed their willingness to try it as opposed to only 16.4% of vegans and vegetarians (see Table 2 below).

**Table 2:** Attitudes to consuming cultivated meat, by participants’ diet.

	Participant’s diet		
**Would you eat cultivated meat yourself?**	Omnivorous	Vegan and vegetarian	Total
Yes	40.3^A^	16.4^B^	32.5
	(198)	(39)	(237)
I’m not sure	36.5^A^	18.9^B^	30.7
	(179)	(45)	(224)
No	23.2^A^	64.7^B^	36.8
	(114)	^ ^(154)	(268)
Total	100.0	100.0	100.0
	(491)	(238)	(729)

Survey respondents are grouped according to diet. Willingness to consume meat is shown as percentage and number in parentheses. Superscript letters indicate significant differences within rows (between diet groups) as determined by the two-sided test of equality for proportions where ^ab^ indicates p<0.05 and ^AB^ indicates p<0.001.

This finding is in line with previous research which suggests that meat eaters, rather than vegans and vegetarians, could be the primary market for cultivated meat products for human consumption [37, 40, 55, 56]. Vegans and vegetarians may object to cultivated meat for a number of reasons; for example, they may be unconvinced that cultivated meat is a genuinely harm-free product, perhaps because of the continued need for ‘donor animals’ [57] or product testing [58], or else they may believe that humans should not use animals for food regardless of whether animals are harmed [59]. At the same time, cultivated meat might be more appealing to omnivores, who have a higher degree of meat attachment [42, 60] and would only be willing to give up the consumption of conventional meat if the alternative did not differ in taste or texture from the product they usually consume.

### Pet feeding practices

**Fig 2:**
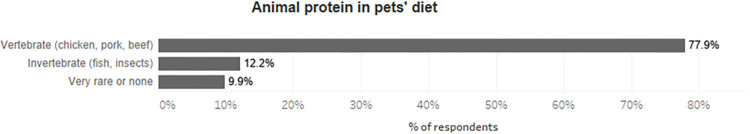
Animal protein in pets’ diet.

As shown in Fig 2, 90.1% of respondents fed at least one of their pets meals consisting of animal protein, with 77.9% of respondents feeding their pets terrestrial vertebrate animals (chicken, beef, pork, game, etc.). Only 9.9% of respondents fed a mostly vegan or vegetarian diet, even though 32.6% of respondents identified as vegan or vegetarian. This indicates that people are more willing to take up meat-free diets themselves than to feed meat-free diets to dogs and cats.

**Fig 3:**
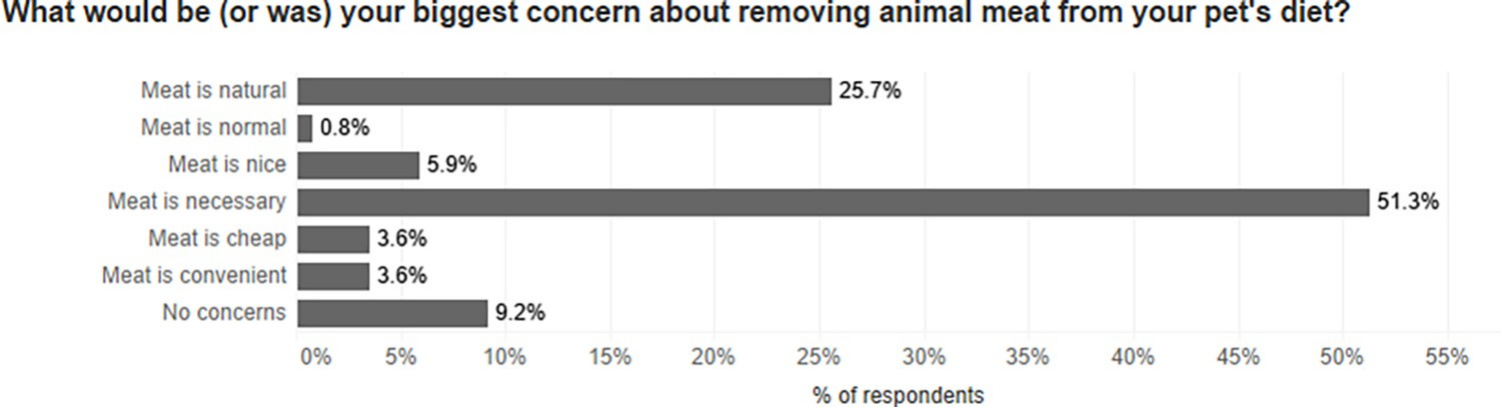
Primary concern about removing meat from pets’ diet. Please note that the figure includes the 3.7% of respondents who believed that meat might be necessary for the health of their pet due to a diagnosed medical condition.

As shown in Fig 3, among the concerns about removing meat from pet diets, 51.3% of respondents selected the ‘necessary’ quality of meat as the greatest concern. This was followed by the ‘natural’ quality, chosen by 25.7% of respondents. Comparatively few chose meat’s ‘niceness’ (5.9%), ‘normality’ (0.8%), price (3.6%), or convenience (3.6%) as their greatest concern.

Table 3 below demonstrates that primary concerns about removing meat from pets’ diets were different for respondents who only owned cats and those who only owned dogs. On average, cat owners were more likely to refer to meat’s naturalness and necessity as their main concern than dog owners. This is not surprising, as cats are obligate carnivores, while dogs are omnivorous. Cat owners were less likely to express no concerns than dog owners, which corresponds to their worries about the appropriateness of meat-free diets for cats [61]. Finally, cat owners were less likely to select the price and convenience of alternative diets as their main reservations. We think a possible explanation for this is that they, unlike some dog owners, are not thinking about the practicalities of meat-free diets. We contend that questions of price and convenience are more likely to enter if plant-based food is a genuine option, rather than an abstract possibility. If we are right, then plant-based pet food is not, for many cat owners among our participants, a real option, even while it might be for many of our dog-owning participants.

**Table 3:** Primary concern about removing meat from pet’s diet, by pet type.

“What would be (or was) your biggest concern about removing animal meat from your pet’s diet?”	Dog owners	Cat owners	Total
**Natural**: my pet is meant to eat meat	20.8^A^	33.9^B^	26.4
	(66)	(81)	(147)
**Normal**: there is societal pressure to feed my pet meat	0.6	0.8	0.7
	(2)	(2)	(4)
**Nice**: I would be unfairly depriving my pet by removing meat	7.9	4.2	6.3
	(25)	(10)	(35)
**Necessary**^1^: I believe it would be a risk for my pet’s health to remove meat from their diet	45.1^a^	55.2^b^	49.5
	(143)	(132)	(275)
**Cheap**: vegan or vegetarian pet food is too expensive	4.7^a^	1.3^b^	3.2
	(15)	(3)	(18)
**Convenient**: it is difficult to find good vegan or vegetarian food	6.0^a^	0.8^b^	3.8
	(19)	(2)	(21)
I have no concerns about removing animal meat from my pet’s diet	14.8^A^	3.8^B^	10.1
	(47)	(9)	(56)
Total	100	100	100
	(317)	(239)	(556)

Survey respondents are grouped according to pet ownership. Concerns about removing meat from a pet’s diet are shown as percentage and number in parentheses. Superscript letters indicate significant differences within rows (between pet owner groups) as determined by the two-sided test of equality for proportions where ^ab^ indicates p<0.05 and ^AB^ indicates p<0.001.

Please note that 173 participants who owned both cats and dogs were excluded from the analysis.

^1^ Please note the table includes 3.7% respondents who believed that meat might be necessary for the health of their pet due to a diagnosed medical condition

To investigate concerns about removing meat from pets’ diets further, we disaggregated them by participant’s dietary choice. We divided the population into omnivores and vegetarians/vegans. As shown in Table 4 below, the biggest concern for vegans and vegetarians was similar to that of omnivores–that meat is necessary (46.2% of vegans/vegetarians and 53.7% of omnivores) for their pets. However, vegans and vegetarians were much less likely to identify concern over the ‘naturalness’ of meat for their pet(s) as their main reservation. A much greater proportion of vegans and vegetarians chose concerns about the price (7.14%) and convenience (8.40%) of vegan or vegetarian pet food as their primary reservation, compared to omnivorous respondents (1.83% and 1.22% respectively).

**Table 4:** Primary concern about removing meat from pet’s diet, by respondents’ diet.

	Respondents’ diet		
“What would be (or was) your biggest concern about removing animal meat from your pet’s diet?”	Omnivores	Vegans and vegetarians	Total
**Natural**: my pet is meant to eat meat	29.74^A^	17.23^B^	25.65
	(146)	(41)	(187)
**Normal**: there is societal pressure to feed my pet meat	0.41	1.68	0.82
	(2)	(4)	(6)
**Nice**: I would be unfairly depriving my pet by removing meat	5.09	7.56	5.9
	(25)	(18)	(43)
**Necessary**^2^: I believe it would be a risk for my pet’s health to remove meat from their diet	53.77	46.22	51.3
	(264)	(110)	(374)
**Cheap**: vegan or vegetarian pet food is too expensive	1.83^A^	7.14^B^	3.57
	(9)	(17)	(26)
**Convenient**: it is difficult to find good vegan or vegetarian food	1.22^A^	8.4^B^	3.57
	(6)	(20)	(26)
I have no concerns about removing animal meat from my pet’s diet	7.94	11.76	9.19
	(39)	(28)	(67)
Total	100	100	100
	(491)	(238)	(729)

Survey respondents are grouped according to diet. Concerns about removing meat from a pet’s diet meat are shown as percentage and number in parentheses. Superscript letters indicate significant differences within rows (between diet groups) as determined by a two-sample t-test where ^ab^ indicates p<0.05 and ^AB^ indicates p<0.001.

^2^ Please note the table includes 3.7% respondents who believed that meat might be necessary for the health of their pet due to a diagnosed medical condition.

### What is the relationship between consuming cultivated meat and feeding cultivated meat?

**Fig 4:**
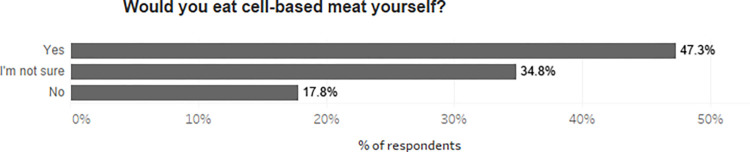
Willingness to feed cultivated meat.

Among all respondents, 32.5% stated that they would eat cultivated meat themselves (see Fig 1), yet a much higher number– 47.3%–said that they would be willing to feed it to their pet(s) (Fig 4 below).

The relationship between willingness to feed and willingness to eat is investigated in Table 5. It shows that a large majority of those who were willing to consume cultivated meat were also willing to feed it to their pets (81.4%). This is expected, since individuals are likely to have a high standard for the food they consume. If they deem it healthy and palatable for themselves, they are also likely to judge it as suitable for their companion animals. The remaining respondents in this category mostly indicated that they were not sure (16.5%), and only a very small minority would eat but not feed the product (2.1%). Similarly, respondents who were not sure about eating cultivated meat were also most likely to be unsure about feeding it (67.3%). The most interesting finding, however, is that among those not willing to consume cultivated meat, a similar proportion of individuals was willing to feed it (36.2%) and not willing to feed it (39.9%). This suggests that the market for cultivated meat for human consumption might be different than the market for cultivated pet food, and that personal attitudes to cultivated meat are not always a good predictor of consumer behavior in relation to pet food.

**Table 5:** The relationship between the willingness to consume cultivated meat and the willingness to feed cultivated meat to pets.

	Would you eat cultivated meat yourself?					
Would you feed cultivated meat to your pets?	Yes	All other responses	I’m not sure	All other responses	No	All other responses
Yes	81.4^A^	18.6^B^	24.6^A^	75.5^B^	36.2^A^	63.8^B^
	(193)	(44)	(55)	(169)	(97)	(171)
I’m not sure	16.5^A^	83.5^B^	67.4^A^	32.6^B^	23.9^A^	76.1^B^
	(39)	(198)	(151)	(73)	(64)	(204)
No	2.1^A^	97.9^B^	8.0^A^	92.0^B^	39.9^A^	60.1^B^
	(5)	(232)	(18)	(206)	(107)	(161)

Survey respondents are grouped according to willingness to eat cultivated meat. Feeding preferences are shown as percentage and number in parentheses. Superscript letters indicate significant differences within rows (between levels of willingness to feed cultivated meat to pets) as determined by the two-sided test of equality for proportions where ab indicates p<0.05 and AB indicates p<0.001. Three separate tests of quality were performed in Table 5 –one for each of the three columns. The test in the first column compares those willing to eat cultivated meat against all other responses. The test in the second column compares those unsure about eating cultivated meat against all other responses. The test in the third column compares those not willing to eat cultivated meat against all other responses.

To find out why this might be the case, we disaggregated the results by participants’ dietary choices. As shown in Table 6, the greatest discrepancy between the willingness to consume and to feed cultivated meat is seen among vegetarians and vegans. Unlike omnivores, most vegans and vegetarians unsure about eating cultivated meat and unwilling to eat cultivated meat were nonetheless willing to feed cultivated meat to their pets (62.2% and 55.8% respectively, compared to 15.1% and 9.6% among omnivores). These are significant differences, demonstrating the important effects of dietary choices on potential for interest in cultivated pet food.

**Table 6:** The relationship between the willingness to feed cultivated meat and consume it, by respondents’ diet.

		Diet	
Would you eat cultivated meat yourself?	Would you feed cultivated meat to your pet(s)?	Omnivores	Vegetarians and vegans
Yes	Yes	78.8^a^	94.8^b^
		(156)	(37)
	I’m not sure	18.7^a^	5.1^b^
		(37)	(2)
	No	2.5	0
		(5)	(0)
I’m not sure	Yes	15.1^A^	62.2^B^
		(27)	(28)
	I’m not sure	75.4^A^	35.6^B^
		(135)	(16)
	No	9.5	2.2
		(17)	(1)
No	Yes	9.6^A^	55.8^B^
		(11)	(86)
	I’m not sure	17.5^a^	28.5^b^
		(20)	(44)
	No	72.8^A^	15.6^B^
		(83)	(24)

Survey respondents are grouped according to diet and willingness to consume cultivated meat. Feeding preferences are shown as percentage and number in parentheses. Superscript letters indicate significant differences within rows (between diet groups) as determined by the two-sided test of equality for proportions where ^ab^ indicates p<0.05 and ^AB^ indicates p<0.001

### What were the main reservations about feeding cultivated meat?

Table 7 demonstrates that among those who said they *would* feed cultivated meat, the greatest proportion of respondents indicated that they had no concerns about it (44.9%). Another 23.5% indicated that their primary concern was that it would be expensive, and 22% were primarily worried about the health and safety implications of the product. The most frequent primary concern among those *unwilling* to feed cultivated meat was that the product was ‘unnatural’ (43.9%). A primary concern that cultivated meat is unnatural seems to be associated with its acceptance (or lack of acceptance) by pet owners–only 4% of those willing to feed it to their pets named its putative unnaturalness as their main reservation. This increased to 15% among those who were not sure and raised sharply to 43.9% among those *not* willing to feed it.

**Table 7:** Willingness to feed cultivated meat, by main reservation about feeding cultivated meat to pets.

	Would you feed cultivated meat to your pet?					
Main reservation about feeding cultivated meat	Yes	All other responses	I’m not sure	All other responses	No	All other responses
It’s unnatural	4.1^A^	24.7^B^	15.0	15.0	43.9^A^	8.7^B^
	(14)	(95)	(38)	(71)	(57)	(52)
I am worried about my pet’s safety / health	22.0^A^	39.1^B^	46.9^A^	22.5^B^	23.9	32.6
	(76)	(150)	(119)	(107)	(31)	(195)
I’m worried they won’t like it	0.6	0.8	0.4	0.8	1.5	0.5
	(2)	(3)	(1)	(4)	(2)	(3)
I’m worried about the ethics of cultivated meat	1.5	1.0	1.2	1.3	0.8	1.3
	(5)	(4)	(3)	(6)	(1)	(8)
I don’t think it would be more environmentally friendly	0.0	0.3	0.4	0.0	0.0	0.2
	(0)	(1)	(1)	(0)	(0)	(1)
It’s expensive	23.5^A^	5.0^B^	7.5^A^	17.1^B^	0.0^A^	16.7^B^
	(81)	(19)	(19)	(81)	(0)	(100)
My pet is fine eating plant-based alternatives	0.3^A^	6.5^B^	3.9^a^	3.4^b^	11.5^A^	1.8^B^
	(1)	(25)	(10)	(16)	(15)	(11)
My pet is fine eating a meat diet	1.7^A^	13.5^B^	11.4	6.1	17.7^A^	5.8^B^
	(6)	(52)	(29)	(29)	(23)	(35)
I would need more information / to do more research	1.5^a^	4.7^b^	6.7^A^	1.3^B^	0.8	3.7
	(5)	(18)	(17)	(6)	(1)	(22)
No reservations	44.9^A^	4.43^B^	6.7^A^	32.6^B^	0.0^A^	28.7^B^
	(155)	(17)	(17)	(155)	(0)	(172)

Survey respondents are grouped according to willingness to feed cultivated meat to their pets. Concerns about cultivated meat are shown as percentage and number in parentheses.

Superscript letters indicate significant differences within rows (between levels of willingness to feed cultivated meat to pets) as determined by the two-sided test of equality for proportions where ab indicates p<0.05 and AB indicates p<0.001. Three separate tests of quality were performed in Table 7 –one for each of the three columns. The test in the first column compares those willing to feed cultivated meat against all other responses. The test in the second column compares those unsure about feeding cultivated meat against all other responses. The test in the third column compares those not willing to feed cultivated meat against all other responses.

Interestingly, while a primary concern for the health and safety of pets on a diet of cultivated meat was relatively similar among those willing and not willing to feed it (22% and 23.9% respectively), almost half (46.9%) of those who were *unsure* about feeding cultivated meat were primarily concerned about health and safety. This indicates that a substantial portion of sampled pet owners could be convinced to feed cultivated meat if research provided some reassurance that this type of diet was nutritionally complete and did not carry any risks for animals that consume it.

Very few respondents noted that they were primarily concerned about their pets’ enjoyment of cultivated meat, which suggests that–if respondents do afford weight to their pets’ enjoyment–few individuals in this sample expect cultivated pet food to taste substantially different to (or at least substantially worse than) conventional pet food. Similarly, very few respondents noted the ethics or environmental impact of cultivated pet food as their primary concern, which suggests that respondents do not perceive cultivated meat production to be as harmful to animals or the environment as the production of conventional pet food.

Those who were unsure about feeding cultivated meat to their pets were more likely to indicate the need for more information on the product as their primary reservation, suggesting (if this tendency is also seen in the population at large) that a successful information campaign could improve the uptake of the cultivated per food among undecided consumers.

Finally, and unsurprisingly, respondents who claimed they were not willing to feed cultivated pet food were more likely to indicate the fact that their companion animal was fine on their current meat or plant-based diet as their key reservation.

In this study, we are particularly interested in the discrepancy between those who would eat cultivated meat and those who would choose to feed it. The choices of those who would feed but not eat it can partially be attributed to their diet. However, it is less clear why some individuals who would eat cultivated meat themselves were unsure about feeding it (16.5%) or would choose not to feed at all (2.1%) (see Table 5).

**Fig 5:**
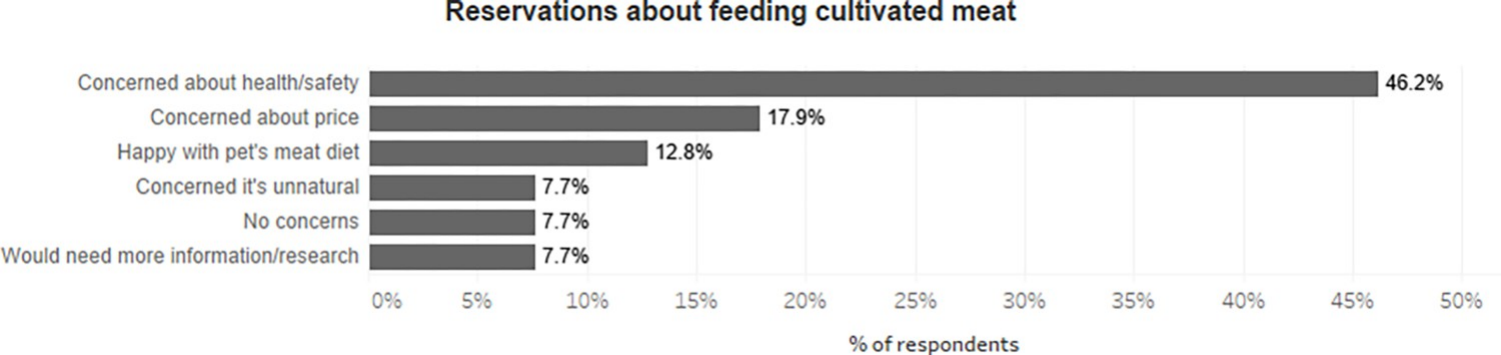
Reservations about feeding cultivated meat among those who would consume themselves, but uncertain if they would feed to their pets (N = 39).

**Fig 6:**
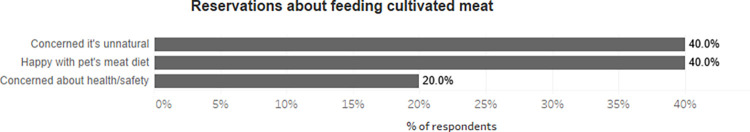
Reservations about feeding cultivated meat among those who would consume themselves, but uncertain if they would feed to their pets (N = 39).

To investigate this further, Figs 5 and 6 describe the main concerns about feeding cultivated meat for those two groups. As shown in Fig 5, those who would eat cultivated meat but have reservations about feeding it most frequently had the safety of the product as their primary reservation (46.2%). We further disaggregated concerns about health between respondents who owned dogs and cats, but the differences in responses were not statistically significant. We think it is possible that some respondents do not perceive cultivated meat as ‘real’ meat and believe that while humans can ‘get by’ on fake meat, companion animals require ‘real’ meat. However, we lack the data to prove or disprove this possibility.

In contrast to the above, the proportion of those who were willing to eat cultivated meat but not feed it was much smaller (2.1%). Fig 6 lists their main concerns, but it is worth noting that the numbers were very small, with only 5 respondents choosing this option. Hence, the results are much less reliable, and we are unable to draw any firm conclusions from the figure. Nevertheless, 2 respondents (40%) indicated that their main reservation was that they were happy with their pet’s meat diet, 2 respondents indicated concerns about ‘naturalness’ of the product as their key reservation (40%), and only 1 respondent (20%) indicated concerns about health and safety of cultivated meat as their primary concern. While no firm conclusions can be drawn, it appears that for those willing to eat but not feed, health and safety concerns are less prominent and ‘naturalness’ might be a much greater concern.

As shown in Table 8 below, vegans and vegetarians were on average more likely than omnivores to state that they do not have any concerns at all about cultivated pet food, which is related to the fact that vegans are also most likely to want to feed cultivated meat. As already highlighted in Table 4, vegans and vegetarians were less likely to express as a primary concern the belief that meat is a ‘natural’ part of companion diet, with only 17.2% of them highlighting as their key concern that their pet is ‘meant’ to eat meat and that removing it would be unnatural, compared to 29.7% of omnivores.

**Table 8:** Main reservations about feeding a cultivated diet to pets, by respondents’ diet.

Main reservation about feeding cultivated meat	Omnivores	Vegans and vegetarians	Total
It’s unnatural	19.6^A^	5.5^B^	15.0
	(96)	(13)	(109)
I am worried about my pet’s safety / health	32.6	27.7	31.0
	(160)	(66)	(226)
I’m worried they won’t like it	0.6	0.8	0.7
	(3)	(2)	(5)
I’m worried about the ethics of cultivated meat	0.2^A^	3.4 ^B^	1.2
	(1)	(8)	(9)
I don’t think it would be more environmentally friendly	0.2	0	0.1
	(1)	(0)	(1)
It’s expensive	12.2	16.8	13.7
	(60)	(40)	(100)
My pet is fine eating plant-based alternatives	0.2^A^	10.5^B^	3.6
	(1)	(25)	(26)
My pet is fine eating a meat diet	11.6^A^	0.4^B^	8.0
	(57)	(1)	(58)
I need more information / to do more research evidence	3.7	2.1	3.2
	(18)	(5)	(23)
No reservations	19.1^A^	32.8^B^	23.6
	(94)	(78)	(172)
Total	100.0	100.0	100.0
	(491)	(238)	(729)

Survey respondents are grouped according to diet. Concerns about removing meat from a pet’s diet meat are shown as percentage and number in parentheses. Superscript letters indicate significant differences within rows (between diet groups) as determined by a two-sample *t*-test where ^ab^ indicates p<0.05 and ^AB^ indicates p<0.001.

Respondents following a more plant-based diet more frequently identified concerns about the ethics of cultivated meat as their primary concern, which may be related to the worry that producing cultivated meat will entail harm to animals or is wrongful regardless of harm to animals. Vegetarians and vegans were also more likely to claim that the fact that their pets were ‘fine’ eating plant-based pet food was their main reservation, while omnivores were more likely to report that their main reservation was that they were happy to keep feeding conventional pet food. This is consistent with the expectation that respondents are more likely to feed their animal in accordance with their own diet, and to be more likely to see this diet as appropriate for their pets. While other differences between omnivores and vegans/vegetarians existed, they were not statistically significant.

**Fig 7:**
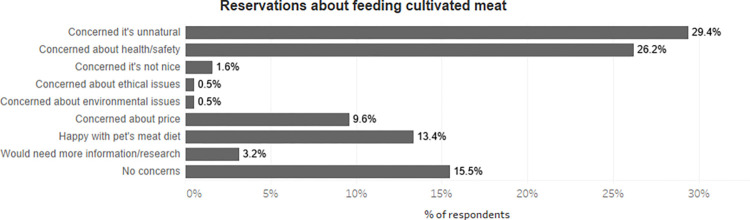
Reservations about feeding cultivated meat among those whose primary concern about removing meat from pet’s diet was that meat was ‘natural’ (N = 187).

Respondents who identified the naturalness of meat-based pet food as their primary worry about switching to plant-based pet food were also the ones most likely to report cultivated meat’s *unnaturalness* as a primary concern, with 29.4% of them choosing unnaturalness of cultivated meat as their biggest reservation for this type of diet (Fig 7). This is interesting because it indicates that appeals to ‘naturalness’ do not serve to justify the feeding of meat as such, but the feeding of meat produced in particular ways.

### Factors affecting the willingness to feed cultivated meat to pets

We performed a set of logistic regressions to establish the factors that are associated with respondents’ willingness to feed cultivated meat to their pet(s) (Tables 9–11). As previously discussed, respondents who were willing to consume cultivated meat themselves were significantly more likely to also feed cultivated meat to their pets. In Table 9, we investigate the importance of the willingness to eat cultivated meat for predicting the willingness to feed it. The effects of many variables, while still statistically significant, become less pronounced when the effects of the willingness to consume cultivated meat is considered. These variables include gender, age, pet diet, and owner diet. The inclusion of ‘willingness to consume cultivated meat’ also greatly improves the model fit and helps explain a greater degree of variance in willingness to feed cultivated meat to pets. This is an important finding because, as established earlier in the paper (Fig 1), over a third of respondents expressed willingness to consume cultivated meat. The findings also suggest that questions about owner food preferences should be included in future survey studies on this topic.

**Table 9:** Factors predicting the willingness to feed cultivated meat to pets.

	Models	
	A1	A2
	OR	OR
	(se)	(se)
Gender (1 = female 0 = male)	0.410***	0.525*
	(0.096)	(0.152)
Ethnicity (1 = white, 0 = ethnic minority)	1.023	0.859
	(0.334)	(0.339)
Age (1 = under 40, 0 = over 40)	1.937***	1.489*
	(0.321)	(0.295)
Pet diet (1 = meat, 0 = no meat)	7.558***	5.901***
	(2.687)	(2.205)
Diet (1 = vegan and vegetarian, 0 = omnivorous)	3.968***	11.623***
	(0.749)	(2.767)
Cat ownership (1 = only cat, 0 = cat and dog/only dog)	0.998	0.867
	(0.173)	(0.181)
Willingness to consume cultivated meat (1 = willing 0 = not sure/not willing)	-	22.085*** (5.374)
Constant	0.130***	0.475***
	(0.065)	(0.027)
N	711	711
Log likelihood	-443.146	-333.181
AIC	900.29	682.36
BIC	932.26	718.89
Pseudo R-squared	0.10	0.32

OR–odds ratio

se–standard error

* 0.01<p<0.05

** 0.001<p<0.01

*** p<0.001

**Table 10:** Factors predicting the willingness to feed cultivated meat to pets–models including primary concerns about removing animal meat from pets’ diet.

	Models						
	B1	B2	B3	B4	B5	B6	B7
	OR	OR	OR	OR	OR	OR	OR
	(se)	(se)	(se)	(se)	(se)	(se)	(se)
Gender (1 = female 0 = male)	0.511*	0.525*	0.525*	0.473*	0.524*	0.523*	0.488*
	(0.15)	(0.15)	(0.15)	(0.14)	(0.15)	(0.15)	(0.15)
Ethnicity (1 = white, 0 = ethnic minority)	0.858	0.802	0.859	0.887	0.855	0.834	0.871
	(0.34)	(0.32)	(0.34)	(0.35)	(0.34)	(0.33)	(0.34)
Age (1 = under 40, 0 = over 40)	1.443	1.502*	1.489*	1.459	1.497*	1.499*	1.510*
	(0.29)	(0.3)	(0.3)	(0.29)	(0.3)	(0.3)	(0.3)
Pet diet (1 = meat, 0 = no meat)	6.331***	6.072***	5.903***	5.271***	5.907***	5.913***	5.135***
	(2.38)	(2.29)	(2.2)	(1.99)	(2.21)	(2.21)	(2.02)
Willingness to consume cultivated meat	21.674***	22.873***	22.099***	21.984***	21.950***	22.015***	22.027***
(1 = willing 0 = not sure/not willing)	(5.31)	(5.61)	(5.38)	(5.4)	(5.34)	(5.35)	(5.36)
Diet (1 = vegan and vegetarian, 0 = omnivorous)	11.130***	12.168***	11.648***	12.229***	11.312***	11.120***	11.575***
	(2.66)	(2.92)	(2.78)	(2.95)	(2.71)	(2.67)	(2.76)
Cat ownership (1 = only cat, 0 = cat and dog/only dog)	0.932	0.847	0.865	0.841	0.882	0.89	0.846
	(0.2)	(0.18)	(0.18)	(0.18)	(0.18)	(0.19)	(0.18)
Meat is natural	0.546**						
	(0.13)						
Meat is normal		0.033*					
		(0.05)					
Meat is nice			0.938				
			(0.37)				
Meat is necessary				1.665*			
				(0.34)			
Meat is cheap					1.652		
					(0.85)		
Meat is convenient						1.822	
						(0.92)	
Other							0.672
							(0.25)
N	711	711	711	711	711	711	711
Log likelihood	-329.70	-329.56	-333.17	-329.95	-332.69	-332.45	-332.62
AIC	677.41	677.12	684.34	677.90	683.39	682.90	683.23
BIC	718.51	718.22	725.44	719.00	724.49	724.00	724.33
Pseudo *R*-squared	0.33	0.33	0.32	0.33	0.32	0.32	0.32

OR–odds ratio

se–standard error

* 0.01<p<0.05

** 0.001<p<0.01

*** p<0.001

**Table 11:** Factors predicting the willingness to feed cultivated meat to pets–models including primary concerns about feeding cultivated meat to pets.

	Models									
	C1	C2	C3	C4	C5	C6	C7	C8	C9	C10
	OR	OR	OR	OR	OR	OR	OR	OR	OR	OR
	(se)	(se)	(se)	(se)	(se)	(se)	(se)	(se)	(se)	(se)
Gender (1 = female 0 = male)	0.524*	0.602	0.528*	0.521*	0.518*	0.518*	0.534*	0.494*	0.520*	0.731
	(0.15)	(0.18)	(0.15)	(0.15)	(0.15)	(0.15)	(0.15)	(0.15)	(0.15)	(0.25)
Ethnicity (1 = white, 0 = ethnic minority)	0.92	0.813	0.865	0.855	0.835	0.814	0.885	0.854	0.888	0.846
	(0.37)	(0.33)	(0.34)	(0.34)	(0.33)	(0.33)	(0.35)	(0.34)	(0.35)	(0.39)
Age (1 = under 40, 0 = over 40)	1.506*	1.487*	1.478*	1.487*	1.483*	1.370	1.43	1.540*	1.518*	1.776**
	(0.31)	(0.3)	(0.29)	(0.3)	(0.29)	(0.28)	(0.29)	(0.31)	(0.3)	(0.39)
Pet diet (1 = meat, 0 = no meat)	6.791***	6.700***	5.931***	5.793***	5.914***	5.420***	5.745***	2.420*	6.177***	4.820***
	(2.57)	(2.55)	(2.22)	(2.17)	(2.21)	(2.04)	(2.16)	(1.03)	(2.32)	(2.00)
Willingness to consume cultivated meat	19.341***	22.540***	22.174***	22.135***	21.968***	19.474***	22.024***	21.888***	22.386***	23.620***
(1 = willing 0 = not sure/not willing)	(4.81)	(5.6)	(5.4)	(5.39)	(5.35)	(4.77)	(5.47)	(5.33)	(5.49)	(6.51)
Diet (1 = vegan and vegetarian, 0 = omnivorous)	9.910***	11.862***	11.653***	11.751***	11.569***	10.510***	10.156***	12.510***	11.735***	10.895***
	(2.41)	(2.88)	(2.77)	(2.81)	(2.75)	(2.52)	(2.44)	(2.99)	(2.81)	(2.97)
Cat ownership	0.815	0.916	0.865	0.869	0.873	0.887	0.854	0.828	0.896	0.897
(1 = only cat, 0 = cat and dog/only dog)	(0.18)	(0.19)	(0.18)	(0.18)	(0.18)	(0.19)	(0.18)	(0.17)	(0.19)	(0.21)
Concerned it’s unnatural	0.179***									
	(0.07)									
Concerned about health/safety		0.396***								
		(0.09)								
Concerned it’s not nice			0.427							
			(0.53)							
Concerned about ethical issues				0.658						
				(0.54)						
Concerned about environmental issues					1.000					
					(0.00)					
Concerned about price						3.385***				
						(1.09)				
Happy with pet’s meat diet							0.150***			
							(0.08)			
Happy with pet’s plant diet								0.039**		
								(0.04)		
Would need more information/research									0.237*	
									(0.14)	
No concerns										18.350***
										(5.99)
N	711	711	711	711	711	711	711	711	711	711
Log likelihood	-320.15	-323.86	-332.93	-333.05	-332.96	-325.07	-325.94	-325.04	-329.85	-277.04
AIC	658.29	665.72	683.87	684.10	681.92	668.14	669.88	668.08	677.71	572.08
BIC	699.39	706.82	724.96	725.20	718.44	709.24	710.98	709.18	718.81	613.18
Pseudo *R*-squared	0.35	0.34	0.32	0.32	0.32	0.34	0.34	0.34	0.33	0.43

OR–odds ratio

se–standard error

* 0.01 < p < 0.05

** 0.001 < p < 0.01

*** p < 0.001

In Tables 9–11, being vegan or vegetarian was also associated with the willingness to feed cultivated meat, even though it was associated with not being willing to consume cultivated meat. Those following more plant-based diets were significantly more likely to be willing to feed their pet(s) cultivated meat, even if other factors normally associated with a vegan or vegetarian diet, such as age or gender, were considered.

Owners of pets on meat diets were significantly more likely to want to feed cultivated meat. This suggests that the market for cultivated pet food is most likely to be found among individuals who currently feed meat to their pets, rather than among those already feeding alternative diets (e.g., plant-based). This finding is particularly striking as, of those who did *not* feed their pets plant-based diets, many in the sample were vegetarian. As we have seen, pet owners who personally follow a plant-based diet were more likely to feed cultured meat relative to an average pet owner in the sample.

In confirmation of our previous discussions, respondents who identified meat’s putative naturalness or normalness for pets as their key reservation about switching to plant-based food were significantly less likely to want to feed cultivated meat. This suggests that the target market for cultivated pet food is among individuals who already feed meat, but perhaps are less emotionally attached to it. At the same time, those for whom their biggest concern about plant-based pet food was that meat is ‘necessary’ were significantly more likely to want to feed cultivated pet food–an indication that cultivated pet food is a useful choice for those who are reluctant to embrace plant-based alternatives for their companion animals.

As shown in Table 11, those whose primary reservations were that cultivated meat products were unnatural, unsafe, or unethical were significantly less likely to want to feed cultivated pet food to their pet(s). Respondents who had no concerns about cultivated pet food and who indicated that the price of cultivated pet food was their biggest concern were significantly more likely to want to feed it to their pets. This is not surprising, since it suggests that those considering cultivated pet food have no reservations about the impact of the product on the health of their pet(s) and are already thinking of practical issues such as price and convenience.

Respondents for whom the primary concern was about cultivated pet food being unnatural or unsafe, or whose primary reservation was that they were happy with their pet’s current diet were (perhaps unsurprisingly) less likely to want to feed cultivated pet food.

Finally, needing more information about cultivated meat was the primary concern for a large proportion of respondents, suggesting that a successful information campaign could improve the uptake of the product.

Being under the age of 40 made people significantly *more* likely to express willingness to feed cultivated meat than older respondents, while female pet owners were significantly *less* likely to do so than male pet owners. However, both effects are only marginally significant, and the effects are not consistent across all models, so these results should be treated with caution.

Interestingly, the type of pet owned by the respondent had no significant impact on their likelihood to feed cultivated meat. It was possible that this result was influenced by the fact that respondents were split into cat owners, dog owners, and dog *and* cat owners, as opposed to exclusive cat or exclusive dog owners. We performed a separate logistic regression on a limited sample of respondents who were either exclusive cat owners or exclusive dog owners, and which included other predictors of willingness to feed cultivated meat (gender, ethnicity, age, participant’s diet, willingness to eat cultivated meat). The impact of owning a cat was still insignificant (OR: 0.73, SE: 0.175, p = 0.189), suggesting that owners of cats and owners of dogs were just as likely to express willingness to feed cultivated meat to their pets.

### Discussion and conclusion

Our results strongly suggest that the market for cultivated pet food differs from the market for cultivated meat for human consumption. Our research, in line with previous findings [37, 55, 56], suggests that when it comes to eating cultivated meat, the main market can be found among omnivores who regularly consume animal protein. However, while willingness to consume cultivated meat and feed cultivated meat were positively correlated, with 81.4% of those in our sample interested in eating cultivated meat willing to feed it to their pets, we observed a large proportion of respondents who were not willing to consume cultivated meat yet still expressed the willingness to feed it to their pet(s). (36.2% of those unwilling to consume cultivated meat themselves in our sample were willing to feed cultivated pet food.) These results suggest a complicated relationship between the market for cultivated meat for human consumption and the market for cultivated pet food.

This complexity is compounded when the possible effect of widespread cultivated pet food on the broader cellular agriculture industry is considered. Naturally, this is something that our data was unable to capture. However, it is worth reflecting on what that impact could be.

On the one hand (and contrary to what was suggested earlier in the paper) introducing cultivated meat to consumers via pet food might lead to feelings of disgust, rather than cultivated pet food being a gateway to cultivated meat for human consumption. People do not think of pet food as suitable for human consumption, and thus an association between pet food and cultivated meat could negatively impact interest in the latter. If that is the case, future research could explore whether the effect could be offset by different kinds of meat being utilized for pet food. For example, perhaps pet food containing cultivated mouse meat (as explored by the startup Because Animals) would be less likely to lead to aversion to cultivated meat, as humans do not see mouse meat as food in the first place. Nonetheless, cultivated pet food containing mouse meat could familiarize consumers with cellular agricultural technologies.

On the other hand, there is increasing consumer interest in so-called ‘human-grade’ food for pets, leading the pet food industry to put increasing emphasis on natural, organic, traceable, sustainable, and certified humane foods. This reflects a humanization in what people are looking for in pet food, and thus, perhaps, a move away from considering foods used for pets as unsuitable for humans. (Indeed, we are aware of no evidence that, for example, dogs and cats being fed beef makes owners less likely to want to eat beef themselves).

In short, further research is needed to clarify the extent to which a fledgling cultivated pet food industry could assist or hinder efforts to introduce cultivated meat for human consumption.

Before detailing further key takeaways from our analysis, it is worth reflecting on limitations of this study. This will allow us to contextualize the results, but also provide directions for future research in this area.

First, as this was a preliminary, exploratory study, the participants did not constitute a representative sample of any real-world population. Future studies could aim for a more representative sample of pet owners in a particular country or region in terms of gender, ethnicity, age, and professional/educational background.

Second, the analysis of motivations/reservations in the present study was limited, as our data concerned only participants’ primary reservations. Thus, it neither illustrated the *range* of reservations that individual respondents had about cultivated or plant-based pet food, nor did it measure the *strength* of individual concerns. Future studies in this area could deploy, for instance, Likert scales to all for more accurate measures of reservations, capturing both the prevalence of varied concerns and their strength.

Third, this study could have done more to distinguish between cat and dog feeding habits. Our data on participants with both cats and dogs (23.7% of the sample) could have better differentiated feeding habits (and concerns) relating to dogs on the one hand and cats on the other. In addition to gathering data from participants about differences between their views and habits concerning dogs and cats respectively, future studies could expand to include other meat-eating companions. Another important study area–captured, to a small extent, by [44]–concerns different sources of cell-based meat. Future studies might explore which different cultivated meats are attractive to potential consumers, and whether companion species makes a difference.

The limitations of the present study acknowledged, our findings suggest that advocates of cultivated pet food should be aware that they cannot assume that openness to cultivated meat for human consumption will translate into willingness to purchase cultivated pet food. Conversely, they cannot assume that aversion to cultivated meat for human consumption will translate into an unwillingness to purchase cultivated pet food. This is an important finding with significant implications for the cultivated meat industry.

The fact that there were respondents who were comfortable consuming cultivated meat but (at least) uncertain about feeding it to their companions may be surprising, and the motivations of this group warrant further research. In this study, such individuals presented a range of primary reasons for being concerned about cultivated pet food. We identified health and safety of the product as one of the most frequent (46.2%), followed by concerns about the price of the product (17.9%). Our data can only take us so far in exploring the basis of a discrepancy between a willingness to eat cultivated meat, but not feed it to companions. However, we offer some speculative indications of what might be at the basis of this discrepancy, stressing that these are proffered as explanations for the existence of a particular group within our data, rather that conclusions that we have read off our data.

First, we acknowledge that health and safety concerns are understandable, given that such products are not yet available, and marketing or information campaigns aimed at alleviating concerns have yet to be conducted. But we add that it is possible that consumers are more willing to take (what they perceive to be) risks with their own health than with their companions’ health. The prevalence of this attitude is attested to by industry surveys. OnePoll, acting on behalf of Spot Pet Insurance, found that 69% of their respondents (made up of 2000 American cat or dog owners) favored their pets’ health over their own [62]. A survey by HealthPocket of American millennials found that 62% of sampled pet owners would take an ill pet to the vet rather than visiting a hospital with their own ailments if they could only afford only one of the two [63].

Incidentally, a willingness among participants to gamble with their own health rather than the health of pets may be particularly true of our sample; as noted, self-selection in our survey (i.e., choosing to participate in a study about pets) may have resulted in a sample of individuals who are particularly concerned with their companion animals’ well-being. For example, as mentioned above, it may have resulted in a sample that is more likely than the general population to consider their pets part of their family. Consequently, our sample might have failed to capture those (a minority, if the aforementioned industry surveys are taken at face value) who are significantly more concerned about their own health than the health of their companion, who might well be willing to favor cultivated pet food if it is cheaper and/or more convenient than conventional pet food, even if they are unsure about its healthfulness.

Second, some consumers may perceive a risk in health for companions while they do not perceive a risk in health for themselves. For instance, if one believes that cats ‘need’ meat, and yet does not believe that cultivated meat is ‘real’ meat, one may believe that cultivated meat will be perfectly healthy for humans, and yet not for cats. As indicated earlier, we cannot test the prevalence of this belief among our participants.

Third, when it comes to price, some consumers may be more willing to pay for (what they expect to be) expensive foods for themselves than for their companions. If cultivated meat is perceived as a luxury, many people may have no intention to feed it to their pets, even if they might try it themselves.

Fourth, notwithstanding the discussion on ‘naturalness’ below, we think it possible that consumers are more motivated to ensure that their companions’ diets are ‘natural’ than they are to ensure their own are, meaning that the (perceived) *un*naturalness of cultivated meat may carry much more weight when it comes to companions’ diets than our own.

Fifth, we suspect that consumers have, in general, only limited awareness of potential problems associated with conventional pet food. While the environmental impact and ethical implications of high-meat *human* diets are now relatively well-known (if not always able to motivate change), the idea that there may be problems with conventional pet food will strike many consumers as something of a novelty–or, even if the possibility is acknowledged, it will not be an issue to which people will devote much thought.

These five possible explanations may account for the group of respondents who were willing to eat cultivated meat, but not feed it to companions. In comparison, there are other concerns with cultivated pet food might be important for those who would not be open to eating cultivated meat themselves–such as concerns about the ethics of cultivated meat or concerns about cultivated meat’s aesthetic merits. But we suspect that these will be less likely to motivate those who are open to eating cultivated meat but not feeding it to their companions. If *these* reasons motivated an aversion to cultivated pet food, they would presumably motivate an aversion to cultivated meat for human consumption, too.

Another significant discovery was that vegans and vegetarians, despite not being willing to consume cultivated meat themselves, were the group most likely to want to feed it to their pets. If this result is generalizable, then perhaps pet food represents an exception to earlier findings that people favor either plant-based diets or cultivated meat, but not both [41]. In fact, consumers might favor plant-based diets for themselves, but cultivated meat for their companions.

Further, that a much greater proportion of vegans and vegetarians, compared to omnivorous respondents, expressed concerns about the price and convenience of vegan or vegetarian pet food suggests that these groups have already considered the possibility of feeding their pets an alternative diet and are thinking of more practical issues. All of this makes vegans and vegetarians a potentially important target market for the cultivated pet food industry. The growing demographic of vegans and vegetarians could provide an important initial market for the producers of cultivated pet food, even though they are unlikely to provide a significant early market for the producers of cultivated meat for human consumption. This also means that there is potential for cultivated meat products to become popular among pet owners, regardless of their own dietary choices.

Key obstacles to switching to cultivated meat for humans often rest upon emotive or non-falsifiable beliefs. For example, previous research shows that people feel disgusted by cultivated meat [64, 65] and believe it to be unnatural [65–67]. As noted by Verbeke et al. [35], things considered to be natural have a highly emotive appeal, evoking feelings of purity (meat produced naturally, not manipulated or changed by humans) and nostalgia (attachment to cooking traditions, memories of eating meat during childhood). It is therefore understandable that many people might feel reluctant to try cultivated meat and might even be disgusted by it.

This finds resonance in how people feed their pets. Of those in this study who listed conventional meat’s naturalness for their pets as their major reservation about plant-based pet food, ‘cell-based meat is unnatural’ was the most frequently chosen primary concern when it came to feeding cultivated pet food. No other group had such a high rate of choosing that answer. This suggests a close connection between highly valuing conventional meat’s putative naturalness for pets (relative to plant-based pet food) and being highly concerned about cultivated meat’s putative *un*naturalness for pets.

Several papers have looked at ways to frame cultivated meat for human consumption to combat perceptions of unnaturalness, such as exposing conventional meat as unnatural [68] or emphasizing the similarities cultivated meat has with conventional meat [69]. However, given that the concept of ‘natural’ or ‘unnatural’ is a rationalization based on affective mechanisms rather than analytic reasoning [70], this group of consumers is likely to be hard to convince.

In fact, arguing for the comparative ‘naturalness’ of cultivated meat for pets may be unnecessary: when it comes to pets, although the perception of conventional meat as natural exists for owners, the pets themselves will not have a cultural or emotional attachment to conventional meat. Very few respondents in our survey expressed questions about cultivated meat being unpalatable or not ‘nice’ for their pets as a primary concern, reflecting, perhaps, their own recognition that the pets themselves would not appreciate the difference.

A large proportion of people in our sample who were undecided about the benefits of cultivated meat for their pets were primarily concerned about the impact of the product on the health of their companion animals. If these numbers reflect broader trends, then a substantial portion of the population could be convinced to feed cultivated meat if research or information campaigns provided some reassurance that this type of diet were nutritionally complete and did not carry any risks for animals that consume it. Given that these consumers are already somewhat open to cultivated pet food, they may represent an ‘easy win’ for the cultivated pet food industry (and perhaps, therefore, the broader cultivated meat industry). This result, however, must be interpreted with caution. Given that some of our participants were recruited through health-focused social media groups, it is at least possible that our respondents are more concerned about health than the general population.

On the other hand, the results suggest–again, if our sample reflects broader population trends–that there is comparatively little to be gained in stressing pets’ enjoyment of cultivated pet food, as few respondents noted this issue as their primary concern.

The results of this study echo those of others [27, 61] about greater willingness of owners to feed a plant-based diet to dogs than to cats. This perhaps reflects the different ethical issues at stake when it comes to feeding plant-based foods to omnivorous dogs than to carnivorous cats [19]. However, what is novel is that there is no significant difference in the willingness of owners of dogs and cats to feed cultivated meat. Thus, contrary to any expectations that may arise from arguments presenting cultivated meat as particularly useful as an ethical means to feed cats [32], and despite the apparent greater prevalence of dogs currently fed a plant-based diet, our results show no reason to believe that cultivated pet food for cats is more likely to be successful than cultivated pet food for dogs.

In our sample, only a very small proportion of people chose to feed their pets a plant-based diet, despite almost a third of respondents identifying as vegan or vegetarian. This indicates that people are more willing to take up plant-based diets themselves but might be reluctant to feed it to their pets. This is likely to create a dilemma for those who abstain from meat, or otherwise recognize the problems of conventional meat production–on one hand, they are likely to have ethical objections to animal agriculture, yet on the other hand they are not willing to feed their companions a plant-based diet because they see it as a necessary part of their nutrition [18, 19]. We contend that cultivated meat has the potential to solve this dilemma by providing a nutritionally adequate food that is not associated with the ethical problems of farmed meat, yet still satisfying the (perceived) nutritional needs of pets. Our results suggest that there are consumers ready to embrace this solution–though further work needs to be done to understand precisely how many such consumers there are, and what steps could be taken to grow their number.

## Supporting information

S1 Dataset Dataset on attitudes to cultivated meat among pet owners. (XLSX)

S1 Data Data key for the dataset on attitudes to cultivated meat among pet owners. (DTA)

S1 Questionnaire Survey questionnaire used in the study. (DOCX)
