# Supplementary material for: Investigating the market for cultivated meat as pet food: A survey analysis
Source: PLoS One. 2022 Dec 30;17(12):e0275009. doi: 10.1371/journal.pone.0275009 (PMC9803157; doi:10.1371/journal.pone.0275009)
Supplement: S1 Questionnaire — (DOCX) [file pone.0275009.s003.docx]

**Survey questionnaire**

**Dog and cat owner attitudes towards feeding their pet(s) meat alternatives**

**Part One – Demographic data**

**These questions will establish some basic information about you and your pet(s).**

1. **What gender do you identify as? (single choice)**
2. Male
3. Female
4. Other
5. Prefer not to say
6. **What is your age? (single choice) Please note that this survey is only for those over 16 years of age.**
7. 16-20
8. 21-30
9. 31-40
10. 41-50
11. 51 -60
12. 61-70
13. Over 71
14. **What county/state and country do you live in? i.e., London, UK or Oregon, USA.**

**______**

1. **What is your profession? Specify in one line.**

______

1. **What is your ethnicity?**
2. White
3. Mixed / Multiple ethnic groups
4. Asian / Asian British
5. Black / African / Caribbean / Black British
6. Other ethnic group
7. Prefer not to say
8. **Do you identify as vegan (consume no animal meat, dairy, eggs or animal products), vegetarian (consume no land animal meat or fish), pescatarian (consume no land animal meat but do eat fish), meat-reducer (cutting back on animal meat consumption), or omnivore (consume most animal meats)? (single choice)**
9. Vegan
10. Vegetarian
11. Pescatarian
12. Meat-reducer
13. Omnivore
14. **What sort of pet(s) do you own? Tick all that apply.**
15. Cat(s)
16. Dog(s)
17. **Which of these statements *most* accurately describes how you see your pet(s)? (single choice)**
18. Like a child to me
19. Part of my family
20. A valued companion
21. A cute addition to my household
22. Protection
23. A working animal (therapy animal/Guide Dog/other)
24. Just a pet

**Part Two – How you feed your pet(s)**

**The next set of questions relate to your existing feeding practices: the food or diet that you currently feed your pet(s).**

**Note: For the purposes of this survey, ‘animal meat’ refers to vertebrate animals: cows, pigs, chickens, fish – but *not* insects, unless explicitly stated. Animal meat includes meat-based dry kibble/wet canned food and raw meat.**

1. **Is your pet on a specialised medical diet? (single choice)**
2. No.
3. Yes. Please give brief details: ____________
4. **Do you currently feed your pet(s) animal meat? (single choice)**
5. Yes, I feed mostly vertebrate land animal meat like chicken, beef and pork
6. Yes, but mostly fish
7. No, but I feed insect-based food
8. No, none at all or very rarely
9. I have more than one pet and I feed them differently i.e., one eats animal meat, the other does not. Please give specifics: ___________
10. **If you feed one or more of your pets animal meat, how often?** Choose the answer that best applies (single choice) and note again that ‘animal meat’ includes meat-based dry kibble, wet canned food and raw meat.
11. For every or nearly every meal and treat
12. Meat-based main meals (dry kibble, wet food or raw meat) with *meat-free* treats
13. Meat-based treats, but vegan or vegetarian main meals
14. I do not feed my pet(s) any vertebrate animal meat
15. **What statements best describe how you perceive the animal meat in your pet’s food? (Tick all that apply)**
16. I choose it as a high quality or premium ingredient
17. I expect to see it in the ingredients list and would worry if it wasn’t there
18. I expect to see it in the ingredients list and would be surprised if it wasn’t there
19. It makes me feel guilty about other animals or the environment
20. I worry about how healthy/high quality it is for my pet
21. Meat isn’t something I’d feel comfortable feeding my pet so I don’t do it
22. **What brand(s) of food do you feed your pet(s)? If you don’t feed a commercially purchased brand, explain what you feed your pet(s) instead.**

­­______

1. **Do you think there are any problems associated with animal meat-based pet food? (Tick all that apply)**
2. Yes, environmental problems
3. Yes, health or contamination problems
4. Yes, feeding my pet other animals contributes to animal welfare issues/is unethical
5. No, I’ve never considered any of these issues
6. Other: ________________

**Part Three – Alternatives to conventional meat in pet food**

**The final set of questions relate to your views on removing vertebrate meat in your pet’s diet, and replacing this with cell-based (‘lab-grown’) meat or another alternative protein.**

1. **What would be (or is) your primary motivation for removing animal meat from your pet’s diet? (single choice)**
2. Environmental/sustainability concerns
3. Ethical/animal welfare/animal rights concerns
4. Health: I have concerns over food safety or nutritional quality related to meat in pet food
5. Price
6. I see no reason to remove meat from my pet’s diet
7. **What would be (or was) your biggest concern about removing animal meat from your pet’s diet? (single choice)**
8. It would be unnatural (my pet is *meant* to eat meat)
9. Meat is normal (I feel societal pressure to feed my pet meat)
10. Meat is nice for my pet (I would be unfairly depriving my pet by removing meat)
11. Meat is necessary (I believe it would be a risk for my pet’s health to remove meat from their food – there’s not enough nutritional evidence that plant-based pet foods can meet their biological needs)
12. Meat is necessary (my pet has a medical condition that requires a specialized diet and I don’t know how a plant-based regime would sit with this)
13. Price: vegan or vegetarian pet food is too expensive
14. Convenience: it is difficult to find good vegan or vegetarian food
15. I have no concerns about removing animal meat from my pet’s diet
16. Other: __________________________

*Cell-based meat (also called cultured meat, clean meat or in-vitro meat) is real meat which is grown from animal cells without the need to raise animals. It should not be confused with meat substitutes such as soy, since it is real animal meat: it has the same taste, texture, and the same or better nutritional content as conventionally-produced meat (Anderson and Bryant, 2018).*

1. **Would you eat cell-based meat yourself? (single choice)**
2. Yes
3. No
4. Not sure
5. **What would be your main reservation? (single choice)**
6. It’s unnatural
7. I’m worried about safety/health
8. I’m happy with plant-based alternatives and don’t miss meat
9. I don't think it's ethical to use animals for food at all
10. I’m happy with conventional meat
11. I’m concerned it will be expensive
12. I don't have any real reservations
13. Other: _________________
14. **Would you feed cell-based meat to your pet(s)? If you have more than one pet and only some eat meat, answer with regard to the meat-eating pet(s) (single choice)**
15. Yes
16. No
17. Not sure
18. **What would be your main reservation about feeding cell-based meat to your pet(s)? (single choice)**
19. Unnatural
20. I am worried about safety/health
21. I don’t need to, my pet is fine eating plant-based alternatives
22. I’m happy feeding my pet conventional meat
23. I’m concerned it will be expensive
24. I don’t have any real reservations
25. Other: ________________
26. **What would most help convince you to feed cell-based meat to your pet? (single choice)**
27. Veterinary recommendation
28. Friend or family recommendation
29. More established options
30. If it were cheaper than conventional meat
31. More information
32. Other________________

*Cell-based meat is expected to be more sustainable, safer and healthier than conventional meat, because it is not obtained from animals raised on environmentally destructive factory farms and killed in slaughterhouses* *where the risk of bacterial contamination is high. According to the Good Food Institute, cell-based meat is predicted to be three times as efficient as chicken to produce (currently the most efficient conventionally produced land-animal meat). It is also potentially more ethical than intensively farming and killing animals for meat, produced by taking a small sample of animal cells (without harming the donor) and replicating them in a culture outside of the animal.*

*The resulting product is 100 percent real meat, but without the slaughter, antibiotics, E. coli, salmonella or other pathogens, waste contamination.*

1. **Has this information changed your mind about feeding your pet(s) cell-based meat? (single choice)**
2. Yes
3. Maybe
4. No, I still wouldn’t do it
5. No, I already knew about these benefits and felt positive about feeding my pet cell-based meat.
6. **If you *would* now feed your pet(s) cell-based meat, what was/is the most convincing benefit for you? (single choice)**
7. Environmental benefits
8. Health and safety
9. Ethical factors
10. Authenticity of final product
11. N/A: I’m still not convinced
12. **Would you consider feeding your pet(s) any of the other alternative proteins listed below?** Select all that apply. If you have more than one pet, answer for the pet that you currently feed animal meat to (if you do).
13. Insect protein (for example, cricket flour or mealworm)
14. Algal protein
15. Fungal protein (for example, koji)
16. Yeast protein
17. Plant-based protein
18. None of these
19. Other: __________________

**Further comments and focus group opportunity**

1. **If you have any further comments to add on your pet’s diet or cell-based meat, please do so below.**
2. **Finally, would you be interested in taking part in a focus group in late 2019/early 2020, to explore the topics raised in this survey further? In a small group, you would discuss concerns and benefits of animal meat alternatives in pet food with other pet owners, particularly cell-based meat. The group will be held in central London and will last no more than 45 minutes – 1 hour, with refreshments provided.**
3. No thanks.
4. Yes, please email me at ____________________
